# Telomerase reverse transcriptase mediates EMT through NF-κB signaling in tongue squamous cell carcinoma

**DOI:** 10.18632/oncotarget.20888

**Published:** 2017-09-14

**Authors:** Yan Wu, Chunxiang Bian, Chunlin Zhen, Liu Liu, Zhenghong Lin, Muhammad Farrukh Nisar, Mei Wang, Jörg W. Bartsch, Enyi Huang, Ping Ji, Li Yang, Yanhong Yu, Junfeng Yang, Xuemei Jiang, Julia Li Zhong

**Affiliations:** ^1^ Stomatological Hospital of Chongqing Medical University, Chongqing Key Laboratory of Oral Diseases and Biomedical Sciences, Chongqing Municipal Key Laboratory of Oral Biomedical Engineering of High Education, Chongqing, 401147, China; ^2^ The Base of “111 Project” for Biomechanics and Tissue Repair Engineering, Key Laboratory of Biorheological Science and Technology, Ministry of Education, Bioengineering college, Life Science College, Chongqing University, Chongqing 400044, China; ^3^ Department of Neurosurgery, Phillips-University Marburg, Baldingerstr, Marburg 35033, Germany; ^4^ Department of Urology, First People’s Hospital of Yunnan Province, Kunming, Yunnan, 650032, China

**Keywords:** OTSCC, hTERT, EMT, NF-κB, CRISPR/Cas9

## Abstract

Locoregional lymph nodes metastasis in oral tongue squamous cell carcinoma represents one of important and common prognostic factors for poor clinical outcome. The human Telomerase Reverse Transcriptase (hTERT) is one of key players in cancer metastasis and stemness, but its exact function in tongue squamous cell carcinoma remains unknown. Here, we aim to understand the role of hTERT by utilizing the CRISPR/Cas9 gene editing system to deplete hTERT in the SCC-15 cell line. Functional comparison of SCC-15 control and knockout cells (hTERT^−/−^) showed that loss of hTERT suppressed cell proliferation and migration/invasion. Furthermore, hTERT depletion significantly decreased tumorigenesis, including alterations in cellular morphology that areindicative for epithelial-mesenchymal transition (EMT). Mechanistically we demonstrated that the hTERT knockout attenuates NF-κB signaling via a negative feedback regulation in tumorprogression. From these results we propose a novel molecular mechanism of hTERT to promote SCC-15 invasion and metastasis via NF-κB activation. We conclude that targeting hTERT may represent a new therapeutic strategy to improve therapy and survival of tongue squamous cell carcinoma patients.

## INTRODUCTION

Oral tongue squamous cell carcinoma (OTSCC/TSCC) is a common subgroup of head and neck cancers (HNCs), marked by high rates of lymph node metastasis and incidence on the rise [1, 2]. Currently, the rate is over 300,000 cases annually across the globe*,* accounting for about 24% of all HNC cases. Even most patients are diagnosed with locoregional but imperceptible initial symptoms of the disease, the 5-year survival rate still remains less than 50% due to its aggressive invasiveness and resistance to treatments including chemotherapy, radiation or combination [2–4]. However, no drugs are currently available to effectively treat and control OTSCC aggressiveness for both regional and distant metastasis. With this regard, the development of diagnostic, prognostic and therapeutic systems for OTSCC treatment would be a significant advancement and may further help develop successful therapies.

Telomerase is a human ribonucleoprotein reverse transcriptase (hTERT) that synthesizes telomeric DNA by gradual addition of DNA sequence repeats to the 3′ end of DNA strands in the telomeric regions. These telomeric regions are composed of two main subunits: the catalytic protein hTERT and the ribonucleoprotein template hTR [5]. Telomerase, and specifically its hTERT, is hyperactivated in 85–90% of the main cancer types that contribute to enhanced cell proliferation, cell mortality, carcinogenesis and rapid tumor progression, hence it became a well known biomarker for various tumors [6, 7]. However, the exact role of hTERT in OTSCC remains to be understood. Study of hTERT expression is crucial to unravel its regulation of proliferation and metastasis in OTSCCunder various pathophysiological conditions.

Epithelial to mesenchymal transition (EMT), is a vital process and regarded as a fundamental built-in program in the metastatic cascades that regulate motility and invasion of cancer cells [8]. The mechanisms of metastatic cascades include the activation of several transcriptional repressors, especially Snail and Slug, through multiple cellular signaling pathways such as NF-κB, Wnt and Hedgehog [9–11]. Among several critical factors involved in EMT progression, NF-κB is the most important factor that orchestrates the inflammatory process as well as regulates the initiation and development of a series of cancers [12]. In addition, telomerase directly regulates NF-κB-dependent gene expression by binding to the NF-κB p65 subunit and by recruitment to a subset of NF-κB dependent promoters [13]. We herein hypothesize that hTERT can modulate EMT and metastasis by linkage to the NF-κB pathway. Notably, a possible regulation of the NF-κB pathway by hTERT has not been reported.

In the current study, we used Clustered Regularly Interspaced Short Palindromic Repeats (CRISPR) RNA-guided Cas9 nucleases to knock out hTERT gene. This accurate method of gene editing enabled us to study the functional aspects of hTERT in SCC-15 cells. We demonstrated that hTERT knockout inhibits SCC-15 cell proliferation, migration and invasion by inhibiting EMT via deactivation of NF-κB signaling.

## RESULTS

### Upregulation of hTERT in primary OTSCC tissues and OTSCC cell lines

**Figure 1:**
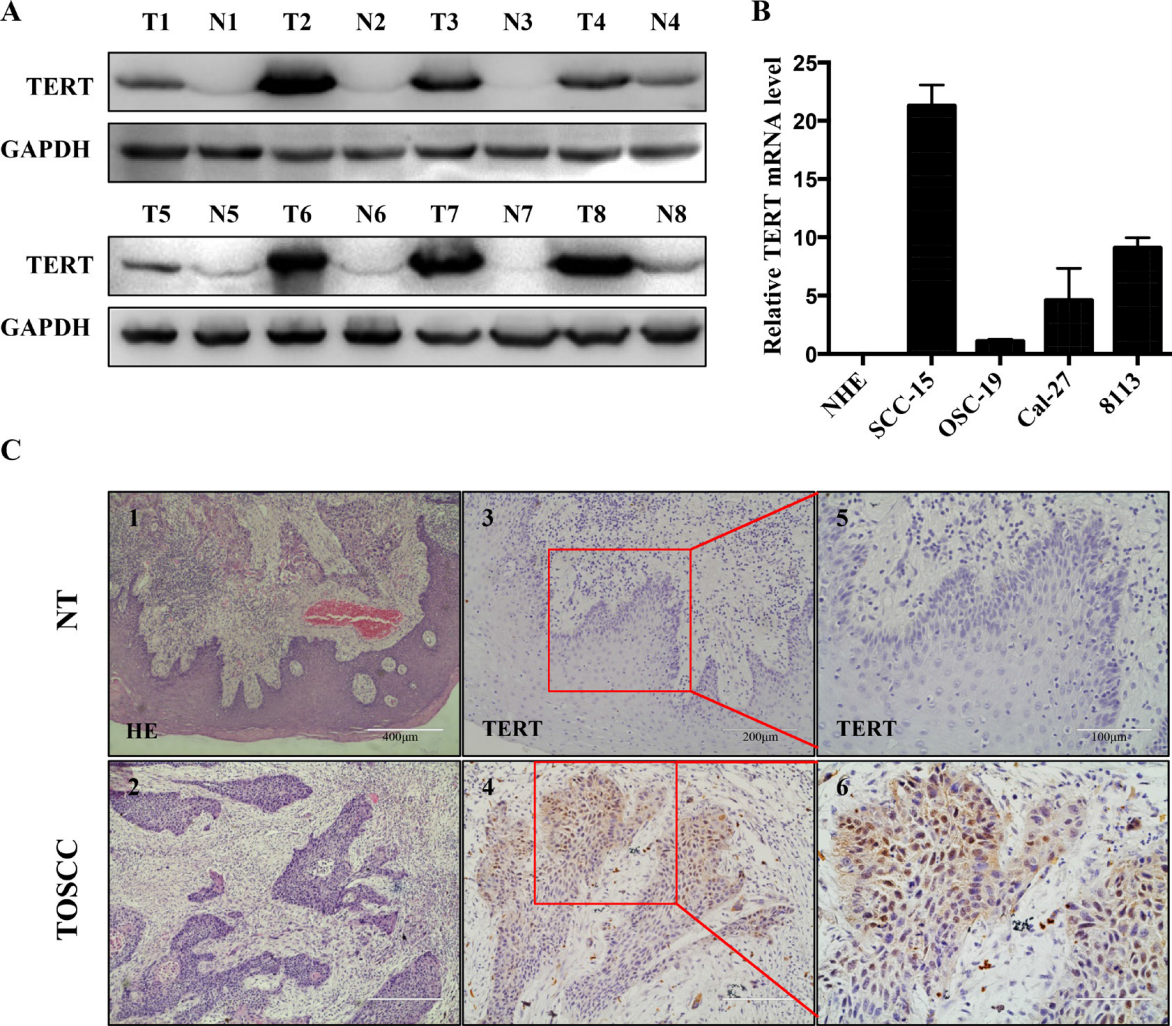
hTERT expression in squamous cell carcinoma tissues and cell lines (**A**) The relative protein expression of hTERT was detected by western-blotting analysis in paired clinical squamous cell carcinoma tissue (T) and adjacent non-tumor tissues (N), with each pair obtained from the same patient. (**B**) RT-PCR analysis of expression of hTERT in different oral squamous cell lines (normal human epithelial (NHE) cells, SCC-15, OSC-19, Cal-27 and 8113). The results are representative of at least 3 replicates. (**C**) HE (1, 2) and immunohistochemical staining (3–6) of paraffin-embedded sections in control group (upper panel) and tongue carcinoma (lower panel) were performed using an antibody against TERT using the streptavidin peroxidase (SP) method. Images 1 and 2, 100 ×magnification, images 3 and 4, 200× magnification, and images 5 and 6, boxed areas in 3 and 4, 400× magnification.

Previous studies revealed that high expression levels of hTERT makes cells highly prone for the development of cancers, as observed in esophageal [15] and stomach carcinoma [16]. To further investigate whether hTERT plays a similar role in the developmentof OTSCC, Western blot and immunoblotting analyses of hTERT expression were performed on paired tongue squamous cell tumor and adjacent non-tumor tissues (N), with each pair obtained from the same patient (Figure 1A). The results confirmed that the protein levels of hTERT were significantly higher in OTSCC samples compared to adjacent non-tumor tissue N, since there was hardly any expression of hTERT detected in the corresponding N tested (Figure 1A). In addition, RT-PCR analyses on tongue squamous cell lines revealed that the expression of hTERT was also remarkably high in SCC-15, Cal-27 and 8113 cell lines, while the expression of hTERT was low in OSC-19 cells, even the hTERT expression could not be detected in NHE cells (Figure 1B). Furthermore, the tissue immunostaining (Figure 1C) revealed that hTERT expression in normal tongue tissues was not detectable. In contrast, hTERT expression was detected as nuclear and cytoplasmic staining pattern in OTSCC tissues. These results showed a notable increase of hTERT in both clinical primary tongue squamous tissues and intongue squamous cell lines.

### Efficient knockout of hTERT in SCC-15 cell lines by the CRISPR/Cas9 system

**Figure 2:**
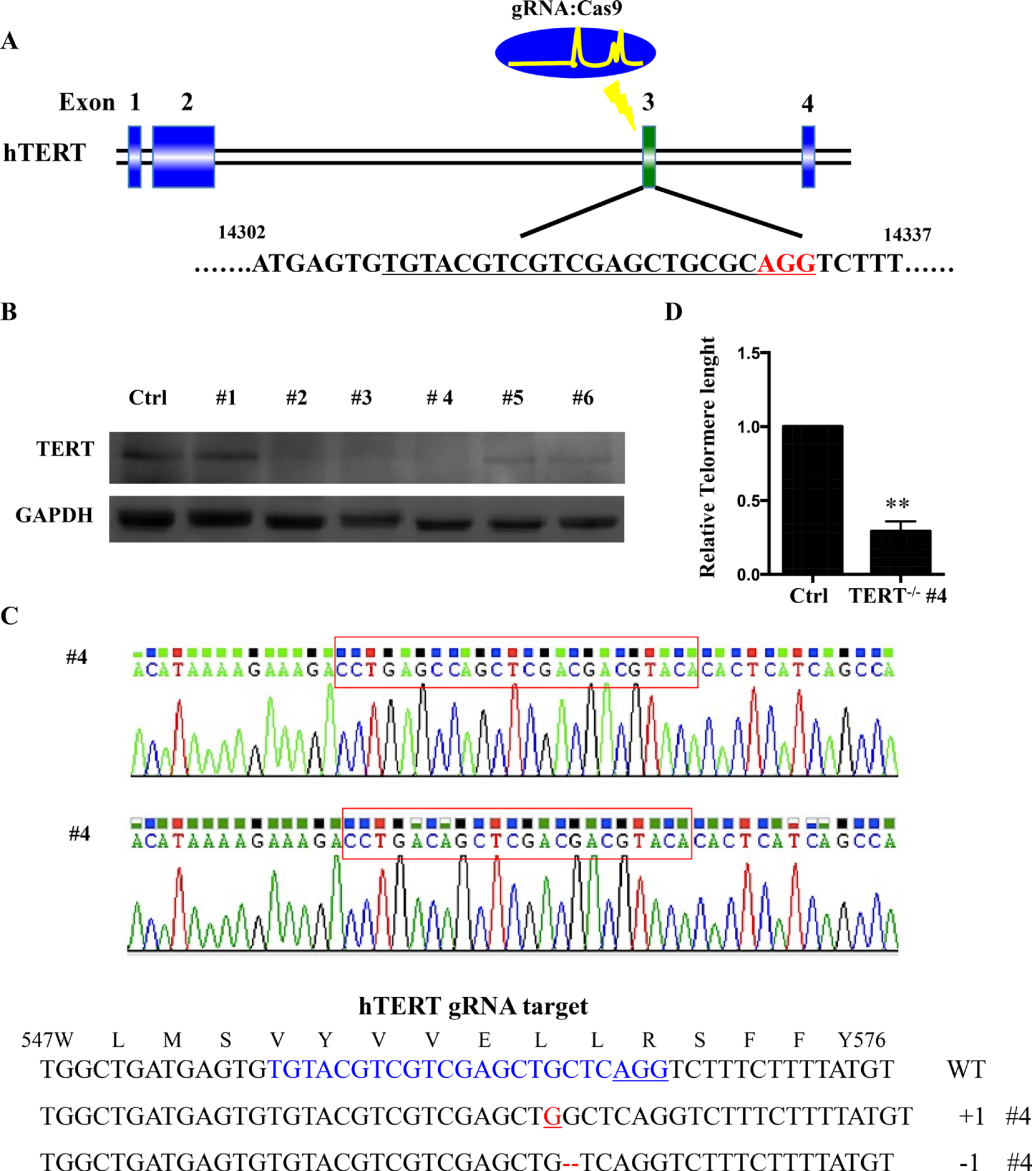
hTERT knockout cells generated using CRISPR-Cas system (**A**) Schematic representation of the genomic DNA structure of hTERT with exons numbered. The sequence including AGG PAM targeted by gRNA in CRISPR-Cas9 system is shown in red. (**B**) Western blot analysis of various knockout cell clones to evaluate the expression of hTERT in squamous cell carcinoma. GAPDH was used as loading control. (**C**) Clone #4 had the highest degree of hTERT reduction and was sequenced and aligned with the wild type sequence (+: inserted bases; −: deleted bases). (**D**) Relative telomere lengths were detected by RT–PCR between hTERT knockout cell line and the control.

We generated a cell line (SCC-15 hTERT KO) with a homozygous knockout of hTERT by targeting exon 3 of hTERT with the CRISPR/Cas9 gene editing system (Figure 2A). SCC-15 cells were infected with lentivirus expressing the designed gRNA and Cas9 protein. One week after infection, cells were reseeded as single cells to obtain single-cell-derived clones. To validate successful hTERT knockout, we performed western blot analysis and target site sequencing. Among 6 clones, we found two homozygous mutant lines and a biallelic 1-nucleotide- inserted and 1- nucleotide-deleted line, #4 (Figure 2B, 2C), and a biallelic 1-nucleotide- deleted and 9- nucleotide-deleted line #3 (Supplementary Figure 1). Comparing with SCC-15 cell line, telomere length is significant decreased in hTERT^−/−^ (#4) measured by quantitative RT–PCR (Figure 2D).

### hTERT knockout suppresses cell proliferation

**Figure 3:**
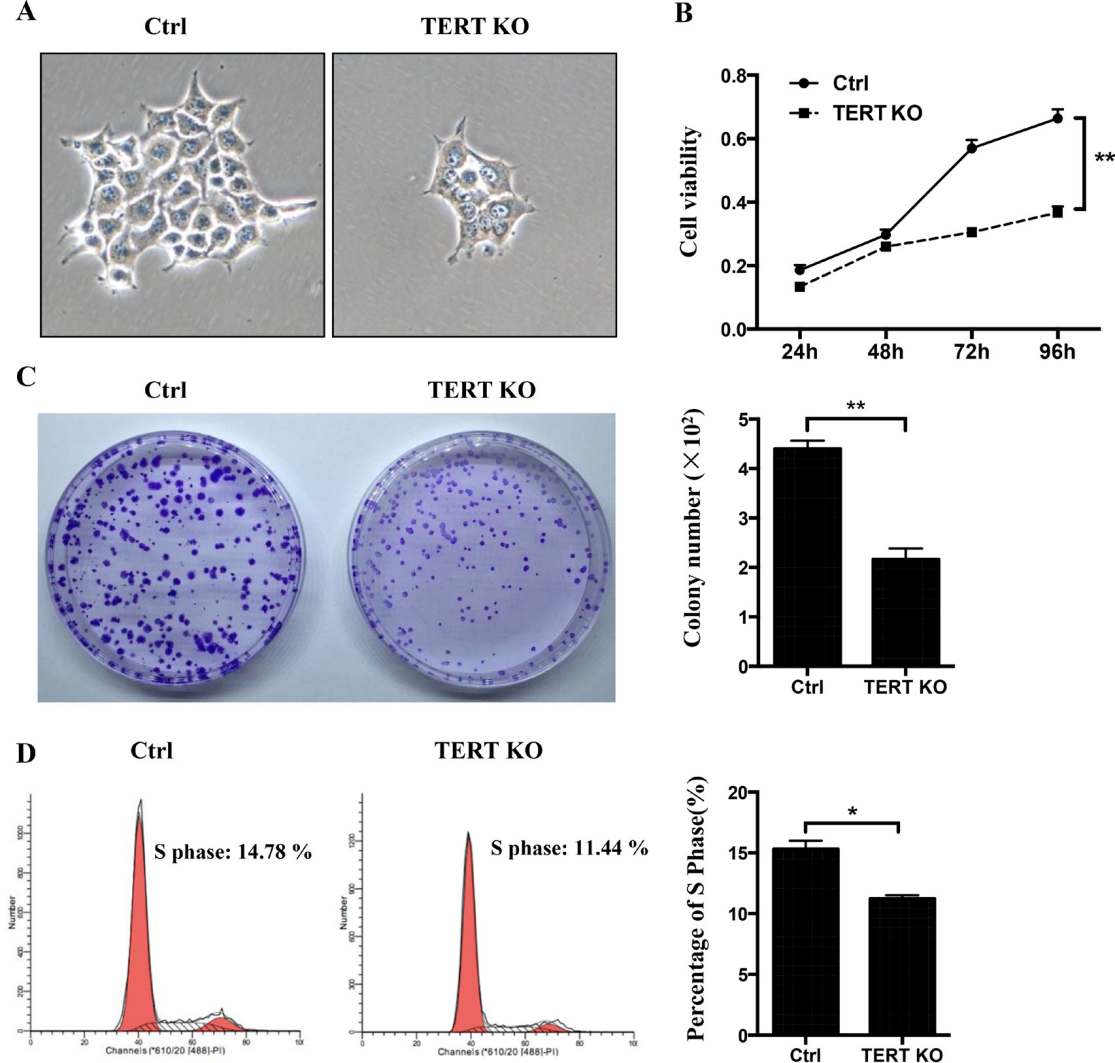
The effects of hTERT on the cell growth in SCC-15 cells (**A**) Morphological changes between the scramble cells and hTERT^−/−^ cells. (**B**) CCK8 assays were used to determine the cell viability after treatment of the hTERT^−/−^ cells. (**C**) The clone numbers of SCC-15 compared with the hTERT^−/−^ cells. Representative pictures (left panel) and a summary graph (right panel) are shown (*n* = 3).(**D**) Effects of knockout of hTERT on cell cycle progression. Representative pictures (left panel) and a summary graph (right panel) are shown (*n* = 3). *P < 0.05; **P < 0.01 vs Ctrl.

Previous studies demonstrated that telomerase activity is necessary for the proliferation of cancer cells, whereas human telomerase reverse transcriptase (hTERT) is an active component of telomerase and is responsible for its catalytic activity [17]. To detect the effect of hTERT on OTSCC proliferation, CCK-8 assay was used to assess cell proliferation and clone formation. The rate and number of proliferation of hTERT^−/−^ cells was significantly reduced compared to control cells (Figure 3A–3C). Consistent with this, the cell proliferation data demonstrate that the hTERT^−/−^ cells population was reduced in the S phase (Figure 3D).

### hTERT knockout inhibits motility and invasion

**Figure 4:**
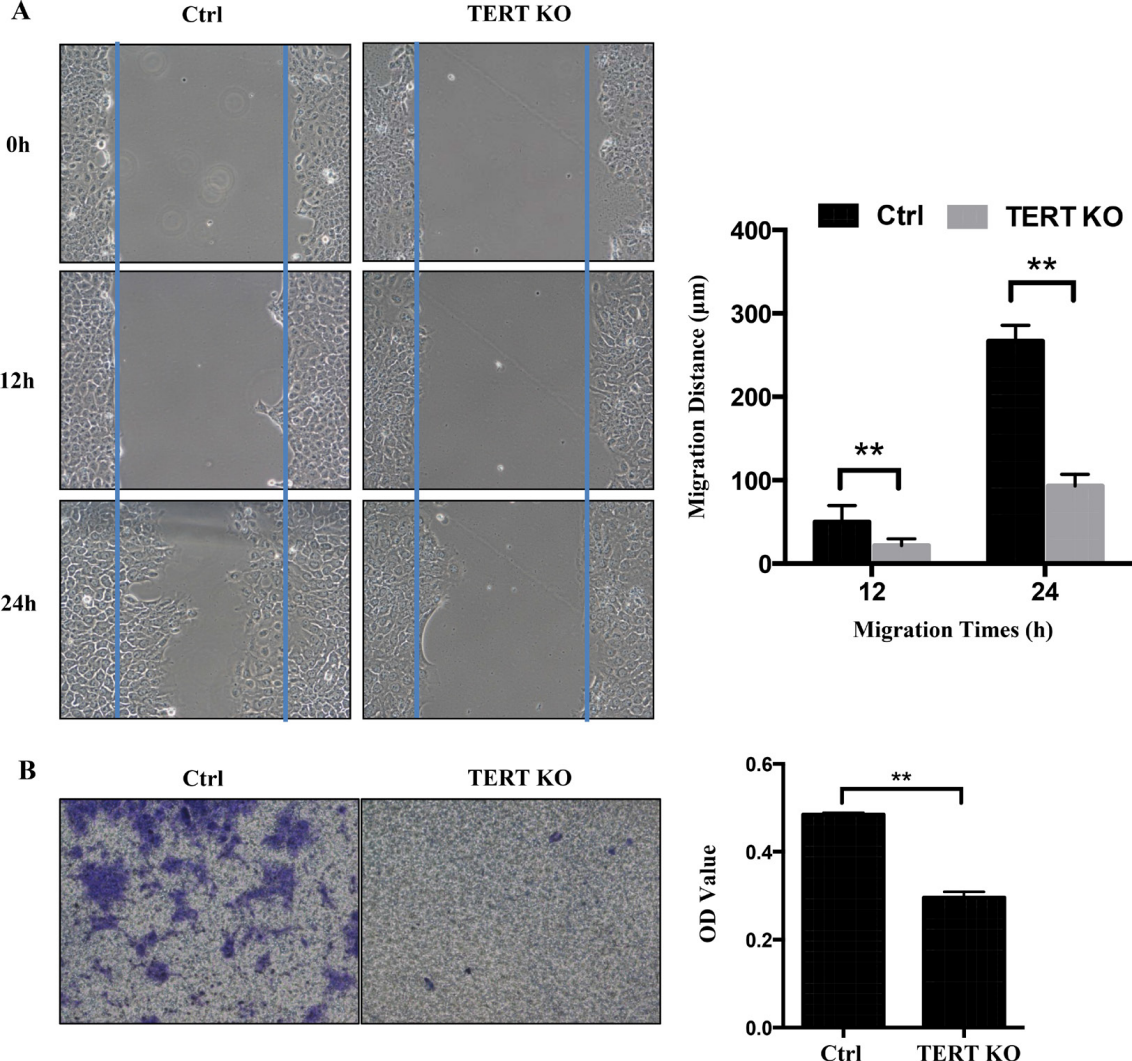
Knockout of hTERT inhibits cell migration in SCC-15 cells (**A**) A confluent layer of control and the hTERT^−/−^ cells was scratched, and the distance of the scratch measured at 0, 12 h and 24 h. Migration distance is shown as the percentage of hTERT^−/−^ cells and the control. (**B**) Transwell motility assays of the hTERT^−/−^ cells and control cells. The number of migrated cells was determined in randomly selected fields and OD value is presented in the bar graph. Error bars represented the mean ± SD of triplicate experiments.**P < 0.05.

Some studies indicated that hTERT expression levels are correlated with tumor stage [18]. These phenomenon led us to hypothesize that hTERT may also be involved in the migration/invasion of OTSCC. To test this hypothesis, cell migration and invasion assays were performed on control and hTERT^−/−^ cells. As expected, wound-healing assay revealed that knockout of hTERT significantly (*P* < 0.05) decelerated wound closure of SCC-15 cells by 60% after 24 h post wounding compared to scramble control cells (Figure 4A). Similarly, knockout of hTERT significantly inhibits invasiveness of SCC-15 cells in Matrigel invasion assays (Figure 4B). These results implicate that hTERT expression in SCC-15 cells can promote cell migration and cell invasion in OTSCC.

### hTERT regulates epithelial-mesenchymal transition (EMT) in SCC-15 cells

**Figure 5:**
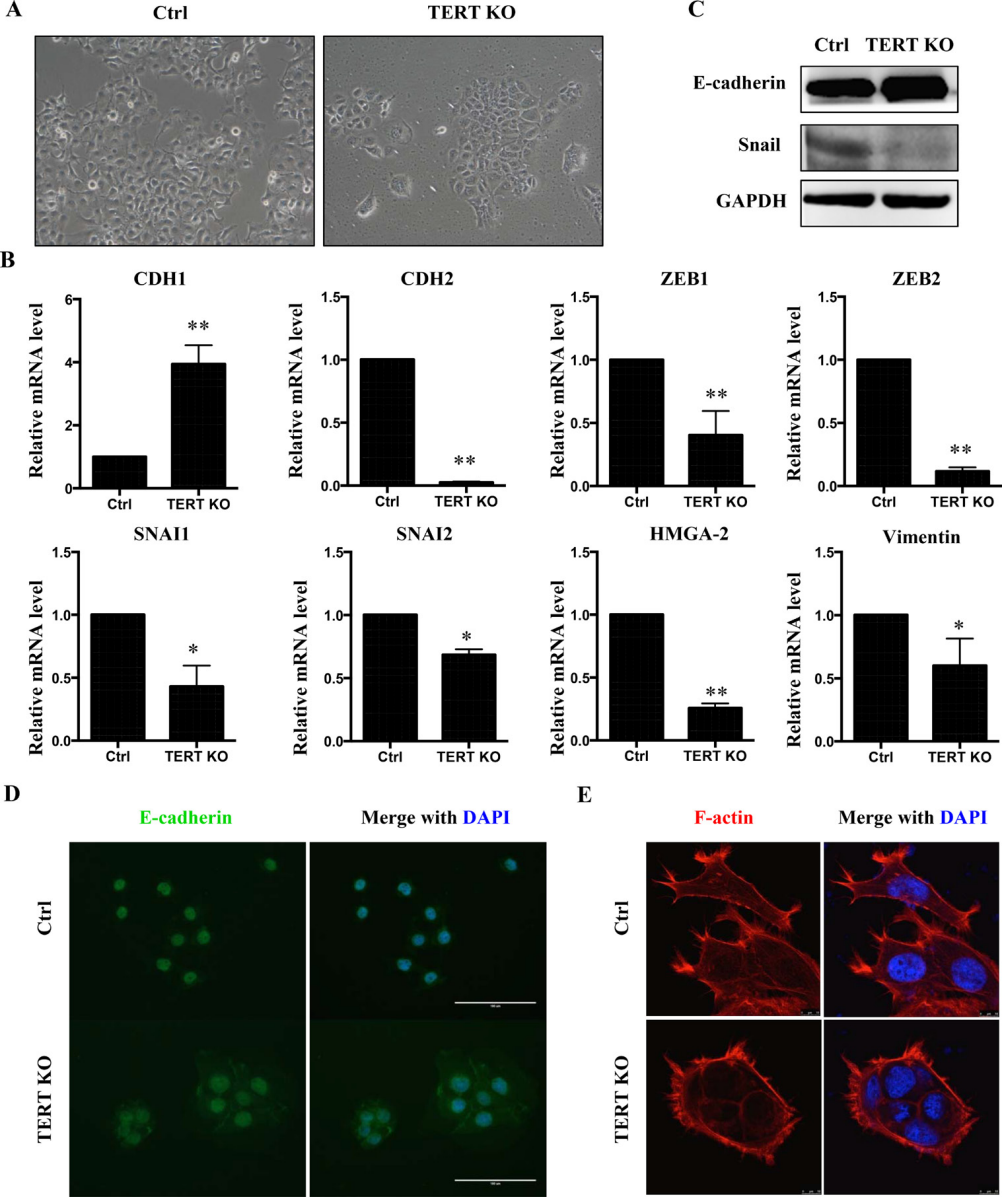
Knock-out hTERT inhibits EMT in SCC-15 cells (**A**) Morphological changes in cells with hTERT knockout compared to control cells. Knockout of hTERT resulted in greater cell-cell contacts when compared with scramble control cells. (**B**) mRNA levels of several EMT makers in scramble and the hTERT^−/−^ cells (*n* = 3). *P < 0.05; **P < 0.01. (**C**) Protein levels of EMT markers, E-cadherin and SNAIL in SCC-15 stable cells assessed by western blotting. (**D**) Fluorescence microscopic staining of e-cadherin (green) is indicated in scramble and knockout cells. Nuclear DNA was stained with DAPI (blue). Scale bar: 100 μm. Data were collected in this set of figures from a representative of at least 3 independent experiments. (**E**) Immunofluorescent staining of F-actin using phalloidin–fluorescein isothiocyanate in scramble and the hTERT^−/−^ cells.

EMT has a critical role in tumor development by reverting epithelial cells to a more mesenchymal state. We therefore analyzed the cellular morphology of SCC-15 cells in more detail (Figure 5A). Scramble control SCC-15 cells display a spindle-like mesenchymal morphology. In contrast, hTERT^−/−^ cells undergo a morphological change to cobblestone-like epithelial cells with more cell-to-cell contacts (Figure 5A). To investigate a potential role of hTERT in the regulation of EMT, a panel of EMT marker genes was examined. Taken together, knockout of hTERT caused an increase in mRNA levels of the epithelial marker CDH1 but a decrease in the levels of mesenchymal markers (Figure 5B). In addition, protein levels of E-cadherin were up-regulated in the hTERT^−/−^ cells, accompanied by a decrease in SNAI1 levels (Figure 5C). Consistent with these results, immunofluorescence staining also showed up-regulation of E-cadherin in hTERT^−/−^ cells (Figure 5D). At the same time, we found that these results in SCC-15 are consistent with results in OSC-19 (Supplementary Figure 2A, 2B). Next, we explored whether an overexpression of hTERT would affect the expression of EMT marker genes in an opposite manner as the hTERT knockout. Overexpression of hTERT (Supplementary Figure 3A) results in decreased levels of CDH1 mRNA, whereas mesenchymal markers SNAI1, SNAI2, ZEB1, VIM and HMG-2 were all increased following hTERT overexpression (Supplementary Figure 3B).

To further investigate the effect of hTERT on EMT, we examined the cellular organization of actin filaments. Using fluorescent staining of actin filaments with phalloidin, a more bundled and circumferential arrangement of the F-actin in the hTERT^−/−^ cells was observed, while the scramble control cells exhibited a more complex array of F-actin, which denotes morphological changes associated with mesenchymal-type cells (Figure 4E).

### hTERT knockout blocks NF-κB signaling pathway

**Figure 6:**
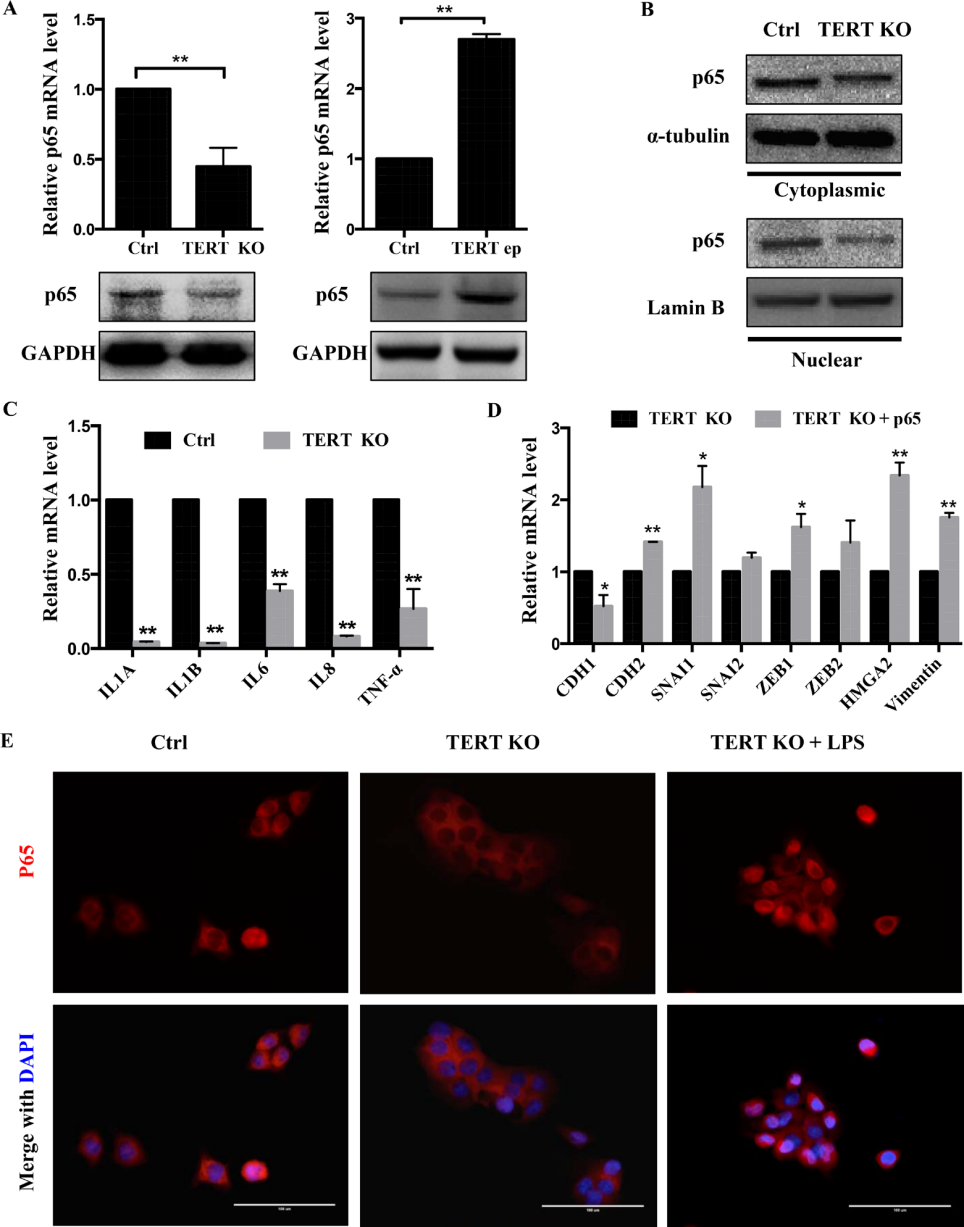
Knockout of hTERT attenuates NF-κB signaling pathway (**A**) mRNA and protein levels of the NF-κB p65 subunit in SCC-15 cells of hTERT over-expression and knockout. (**B**) Knockout hTERT reduced cytosolic and nuclear P65 levels. Cells lysates were prepared from cytosolic and nuclear fractions. P65, GAPDH (cytosolic control) and Lamin B (nuclear control) were analyzed by western blotting. (**C**) The mRNA levels of the NF-κB-dependent gene in SCC-15 stable cells of hTERT knockout. (**D**) Knockout cells were transfected with Flag-p65 or PCMV-Flag controls as indicated. At 48 h later, cells were treated by mRNA extraction and quantitative PCR. Levels of relative EMT mRNA expression for the indicated genes are shown. (**E**) LPS rescues knockout hTERT-dependent inhibition of NF-κB activation. The SCC-15 cells were treated with or without LPS (5 mg/ml) for 4 h. The localization of p65 in SCC-15 cells was determined by immunofluorescence staining. Scale bar: 100 μm. Data were collected in this set of figures from a representative of at least 3 independent experiments. *P < 0.05; **P < 0.01.

Since the NF-κB signaling pathway is known to be involved for invasiveness and metastatic properties of several cancer cell types [19], we sought to investigate the association between the NF-κB pathway and hTERT expression. We first assessed the expression of NF-κB p65 (p65) in hTERT^−/−^ cells and hTERT-overexpressing cells at protein and mRNA levels, and found that knockout of hTERT significantly suppressed the expression of p65 and the nuclear accumulation of p65, while overexpression of hTERT remarkably elevated p65 expression (Figure 6A, 6B). The level of NF-κB-dependent transcription was reduced in hTERT^−/−^ cells (Figure 6C). In addition, overexpression of p65 in hTERT^−/−^ cells (Supplementary Figure 4A) caused a significant increase in the expression levels of a number of endogenous NF-κB targets (Supplementary Figure 4B). Transfection of a construct for p65 overexpression reversed the effect of hTERT knockout on EMT marker expression (Figure 6D). Furthermore, since LPS caused an activation of NF-κB in tumor cells, the subcellular localization of p65 subunit of NF-κB in SCC-15 cells was further examined by immunofluorescence after LPS treatment. The reduction of nuclear translocation of the p65 subunit of NF-κB in the hTERT^−/−^ cells, while it was detected to be highly expressed in the nucleus when treated with LPS by immunofluorescence staining (Figure 6E). Then we studied, whether hTERT knockout inhibited cell invasion was associated with the p65 expression. For this purpose, hTERT^−/−^ cells were transfected with p65 vectors. p65 overexpression rescued the hTERT knock-out phenotype of cell invasion was decreased (Supplementary Figure 4C). Taken together, our results suggest that a knockout of hTERT inhibits NF-κB transcriptional activity and suppresses the EMT, i.e that hTERT promotes NF-κB transcriptional activity and enhances EMT.

## DISCUSSION

Human tongue tumorigenesis is characterized by multiple and consecutive histopathological stages from normal epithelial to invasive OTSCC driven by activation of oncogenes while switching off tumor suppressors. Potent proliferation and metastasis are a frequent cause of death in OTSCC patients, but the underlying mechanisms remain poorly understood. Previous studies suggest that hTERT might act as an oncogene driving tumorigenesis, and hTERT could serve as a novel diagnostic biomarker and therapeutic target for cancer treatment. However, only a few studies have focused on the critical role of hTERT in OTSCC cancer invasion and metastasis [20]. In the current study, the results provide evidence for the notion that hTERT plays a significant role in promoting proliferation and invasion of OTSCC cells.

The current study revealed high expression levels of hTERT in OTSCC tissues compared to normal tongue epithelial tissue. A positive correlation of hTERT levels with the occurrence of OTSCC was observed. This is in agreement with previous studies where high levels of hTERT were detected in esophageal [15], cervical cancer [21], lung cancer [22] and gastric cancer [23]. It was known that hyper-activation of hTERT leads to telomere elongation, which plays a pivotal role in tumorigenesis and metastasis. Conversely, degradation of hTERT by the proteasome or repression of its activity led to telomere shortening, cancer cell apoptosis and even senescence [21, 24]. Human TERT may serve as target in reduction of telomerase as inhibition of hTERT has been shown to suppress telomerase activity and to block cancer cell metastasis effectively by using RNA interference and pharmacological agents e.g. imetelstat (GRN163L) with minimal side effects in phase-1 and -2 clinical trials [25–27]. However, the effects of long-term treatment with telomerase inhibitors have not yet been investigated.

CRISPR-Cas9-mediated genome editing provides an alternative approach, and here we used a CRISPR/Cas9 approach to knock out the hTERT gene in an easy, quick and accurate manner to study the functional consequences of hTERT in SCC-15 cells. We designed sgRNAs for hTERT and cells induced with sgRNA target hTERT over 98%. The hTERT^−/−^ cells showed a typical morphological phenotype (spindle shaped to rounded cell shape change) that was not observed in previous experiments with anti-hTERT where the functional depletion of hTERT might have been insufficient. Knockout of hTERT in SCC-15 cells correlated with telomere length. However, some reports suggested that silencing of hTERT did not modify telomere length in anaplastic thyroid cancer cells [28]. These results suggest that gene silencing by using a siRNA approach is not sufficient for an in-depth study of the molecular mechanisms of hTERT. Thus, we used hTERT^−/−^ cells to comprehensively analyze the role of hTERT and provide strong evidence that hTERT exerts anti-tumor effects in SCC-15 cells. These findings support the notion that hTERT contributes to the development of OTSCC in addition to the effects on cell proliferation, migration and invasion.

Our study disclosed molecular mechanisms by which a genetic loss of hTERT exerts antitumor effects. A number of studies revealed that Epithelial-Mesenchymal Transition (EMT) provides a key role in processes underlying the acquisition of migratory capacity in primary OTSCC cells. Particularly metastasizing OTSCC cancer cells can clearly be distinguished from the core tumor of patients by an expression signature of genes associated with EMT [29]. In addition, various genes that cause EMT are also associated with increased tumor stemness, aggressiveness and chemoresistance [30, 31]. Furthermore, E-cadherin, Snail, and vimentin may function as the key factors in the regulation of epithelial cell-to-cell adhesion [32]. Several experimental and clinical studies have revealed that these factors have been implicated also in pathological alterations of the phenotype associated with the acquisition of invasiveness and cancer stemness by tumors. We demonstrated here that the hTERT^−/−^ cells might induce a pattern of up-regulated epithelial protein (E-cadherin), while down-regulating mesenchymal-like proteins (N-cadherin and Vimentin), which results in tumor metastasis. Finally, we observed a highly bundled and circumferential arrangement of the F-actin in the hTERT^−/−^ cells, while the control cells exhibited a more complex array of F-actin. These findings supported the notion that inhibition of EMT is a major mechanism by which knockout of hTERT inhibits OTSCC cancer development. Most importantly, the metastatic effects of hTERT are associated with the EMT process in OTSCC cells. Our findings offer a new perspective on the role of hTERT in promoting the progression of OTSCC.

Our data further demonstrated that the mechanism of hTERT might involve the suppression of NF-κB signaling. NF-κB is a structurally conserved family of dimeric transcription factors that contain sequences mediating dimerization, DNA binding, nuclear localization, and interaction with inhibitory IκB proteins [33], it plays an important role in both facilitating and maintaining an invasive phenotype [34]. Moreover, numerous sources of evidence have identified NF-κB as an essential mediator of EMT. However, the regulation of hTERT in the NF-κB signaling pathway-induced metastasis and EMT have not been elucidated yet. We confirmed that knockout hTERT blocks the NF-κB signaling pathway. Indeed, our results disclosed that knockout hTERT significantly decreased the translocation of p65 into the nucleus by suppressing NF-κB promoter activity, leading to inhibition of the EMT and migration in OTSCC. While hTERT promotes progression in various stages of carcinogenesis was observed in many studies, down-regulation of hTERT in NF-κB pathway restraining cancer progression was also demonstrated in this study. The hTERT may directly regulate NF-κB activity, but the specific mechanisms driving these interactions in different cancers need further exploration. These results provide a new perspective on the possible underlying mechanisms by which hTERT is involved in metastasis and EMT progression in cancer.

In conclusion, by using CRISPR-Cas9-mediated genome editing we have constructed hTERT knockout cancer cells, and results provide direct evidence for a strong tumor-suppressive effect in hTERT^−/−^ cells. Collectively, SCC-15 hTERT^−/−^ cells inhibit EMT via deactivation of NF-κB signaling, thus hTERT may represent a novel therapeutic target for the treatment of OTSCC tumors.

## MATERIALS AND METHODS

### Tumor samples

Tumor samples from resection specimens were collected from 8 consecutive patients with OSCC, who underwent surgical resection for the disease at College of Stomatology of Chongqing Medical University (Chongqing, China) between August 2015 and December 2016. Informed consent was obtained from the patients. Those patients with preoperative anticancer treatment or with evidence of other malignancies were excluded from the study. The study protocol was approved by the local Ethics Committee of Chongqing Medical University (Chongqing, China).

### Cell growth, proliferation and clonogenic assays.

Stably transfected cells were seeded into 96-well plates (100 μl/well; 3000 cells/well) and incubated at 37°C with 5% CO_2_. 10 μl of CCK-8 solution was added into each well at four-time point followed by incubation for 1h. The change in absorbance at 450 nm was photometrically measured. The experiment was performed three times. The stable cell lines were harvested, centrifuged at 1000 rpm for 3 min and the supernatants were removed. The cell suspension was then fixed in precooled 70% ethanol at 4°C for 24 h. Cell proliferation was detected by flow cytometry. For clonogenic assays, cells were seeded onto 60 mm culture dishes at the density of 1000 cells/well. Cultures were maintained for 9–12 days in complete culture media until colonies appeared. The colonies were fixed with 4% paraformaldehyde and stained with 0.5% crystal violet. The plates were photographed and the numbers of visible colonies were counted.

### DNA extraction and telomere length assay

Extraction of genomic DNA of SCC-15 and hTERT knockout cells was performed as with the GentraPuregene Cell Kit (Qiagen, Hilden, Germany), according to the manufacturer’s protocol. Telomere length was measured with genomic DNA extracted from cell samples, using a real-time quantitative polymerase chain reaction (RT-PCR). The total reaction volume was 25 μl containing 10 ng of genomic DNA, and 12.5 ul GoTaq^®^ qPCR Master Mix (Promega, USA). The final primer concentrations were as follows: for telomere amplification tel1 (5′-GGTTTTTGAGGGTGA -GGGTGAGGGTGAGGGTGAGGGT-3′), 675 nmol/L and for tel2 (5′-TCCCGACTATCCCTATCCCTATCCCTATCCCTATCCCTA-3′), 1350 nmol/L; and for the amplification of the single copy gene RPLPO: hRPLPO1 (5′-CCCATTCTATCATCAACGGGTACAA-3′), 800 nmol/L; hRPLPO2(5′- CAGCAAGTGGGAAGGTGTAATCC-3′), 800 nmol/L. The method normalizes T to S by taking the ratio (T/S ratio) for each sample. The T/S ratio was calculated by [2CT(telomeres)/2CT(single copy gene)] =2-∆CT.

### Generation of knockout cell lines using CRISPR-Cas9 technique

The gDNA for targeting hTERT in lenti CRISPRv2 vector was designed as follow, Oligo 1 5′-CACCGTGTACGTCGTCGAGCTGCTC-3′, Oligo 1 : 3′- CACATGCAGCAGCTCGACGAGCAAA-5′. The lentiCRISPRv2 plasmid harboring the gDNA sequence and Cas9 gene was co-transfected with lentivirus helper plasmids psPAX2 and pCMV-VSV-G into actively growing HEK-293T cells using Lipofectamine 2000 (Invitrogen, USA) according to the manufacturer’s protocol. Virus-containing supernatants were collected respectively 48 and 72 hours after transfection, filtered to eliminate cells and infected target cells in the presence of 8 μg/ml polybrene. 24 h later, infected cells were cultured with 3 μg/ml puromycin for a week. Cells were then replated as single cells at a very low density on 100 mm culture dishes and cultured for ten days with 3 μg /ml puromycin. Individual colonies were picked and expanded. Protein lysates and genomic DNA was then extracted for further analysis.

### Wound-healing and transwell invasion assay

SCC cells were cultured in 6-wells plates and upon 80–90% confluency, cells were treated with 10 μg/ml mitomycin C (Sigma) for 2 h. Using a 200 μL pipette tip, a scratch wound was made in the monolayer. The distance between the two sides of the wound was measured before and after wound repair. The pictures were acquired and further analyzed. The migration distances were measured by software (Nikon) from time 0 to 24 h post scratch. Images were taken of four random optical fields (100×) on each filter. The experiments were conducted in duplicates. Matrigel invasion chamber (8-μm pore size) was used to assess cell invasion ability [14]. Cells were seeded onto the surface of the 24-well Transwell filter inserts (Merck Millipore) (1 × 10^4^cells/well) with medium containing 0.5 % bovine serum albumin, while the medium containing 10 % fetal bovine serum was added to the lower chamber. After 48h of incubation at 37 °C, noninvaded cells on the top of the insert were removed with cotton swabs. The invaded cells were fixed with 4 % paraformaldehyde, stained with 0.1 % crystal violet, and counted.

### RNA extraction and qPCR

Total RNA was extracted using TRIzol reagent (TaKaRa) following the manufacturer’s protocol. RNA was reversed transcribed to cDNA using SuperScriptIII (Promega). qPCR was carried out in 20 μl volumes containing 1× Eva Green qPCR master mix (Applied Biological Materials Inc., Richmond, BC, Canada) on CFX Connect™ Real-Time PCR Detection System (USA). Primers are listed in Supplementary Table 1. Glyceraldehyde-3-phosphate dehydrogenase (GAPDH) was used as an internal control. The relative expression levels of mRNA were quantified using the ΔΔCt method.

### Immunofluorescence and immunohistochemistry

Human skin tissues were embedded in paraffin, 6μm sections were made and stained with TERT antibody (1:100, Abcam) and analyzed by immunohistochemistry methods. Signals were detected by DAB (Zhongshan) staining or directly by fluorescence microscopy. For immunofluorescence, the slides were incubated with E-cadherin antibody (1:200, Cell Signaling Technology), p65 antibody (1:200, Cell Signaling Technology), followed by incubation with FITC-conjugated anti-rabbit IgG and TRITC-conjugated anti-rat IgG, or biotin-conjugated goat anti-rabbit IgG (all 1:100, Zhongshan). Actin filaments were visualized with Rhodamin-Phallodidin (Solabio, China). Slides were observed under a Nikon E600 microscope (Tokyo, Japan) with a digital camera.

### Western blot analysis

Western blotting assay was performed as follows: the protein samples were collected from the human skin tumor or cells. The tissue lysates were separated on 12% SDS-polyacrylamide gels and transferred onto polyvinylidene fluoride membranes (Bio-Rad). Antibodies used included TERT antibody (1:1000), snail antibody (1:1000), E-cadherin antibody (1:1000), while GAPDH (1:2000) was used as a loading control. The Western blot results were further analyzed using BIO-RAD ChenmiDoc™ XRS+.

### Statistical analysis

All values were expressed as mean ± S.D. Statistical analysis of the data was performed by two-tailed Student’s *t* test (*p < 0.05; **p < 0.01).

## SUPPLEMENTARY MATERIALS FIGURES AND TABLE


