# Supplementary material for: Telomerase reverse transcriptase mediates EMT through NF-κB signaling in tongue squamous cell carcinoma
Source: Oncotarget. 2017 Sep 14;8(49):85492–503. doi: 10.18632/oncotarget.20888 (PMC5689625; doi:10.18632/oncotarget.20888)
Supplement: Supplementary file 1 [file oncotarget-08-85492-s001.pdf]

Telomerase reverse transcriptase mediates EMT through NF-κB signaling in tongue squamous cell carcinoma

SUPPLEMENTARY MATERIALS

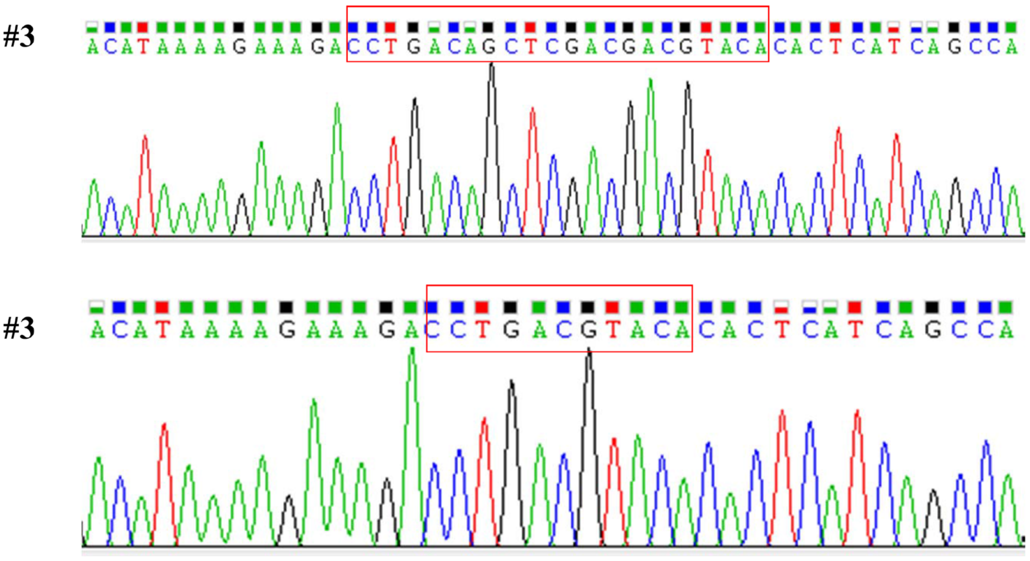

hTERT gRNA target

547W L M S V Y V V E L L R S F F Y576

TGGCTGATGAGTGTGTACGTCGTCGAGCTGCTCAGGTCTTTCTTTTATGT WT

TGGCTGATGAGTGTGTACGTCGTCGAGCTG--TCAGGTCTTTCTTTTATGT -1 #3

TGGCTGATGAGTGTGTACGTCGT-----------------TCAGGTCTTTCTTTTATGT -12 #3

Supplementary Figure 1: The sequencing results of Clone #3. Clone #3 showed the degree of hTERT reduction and was sequenced and aligned with the wild type sequence (−: deleted bases).

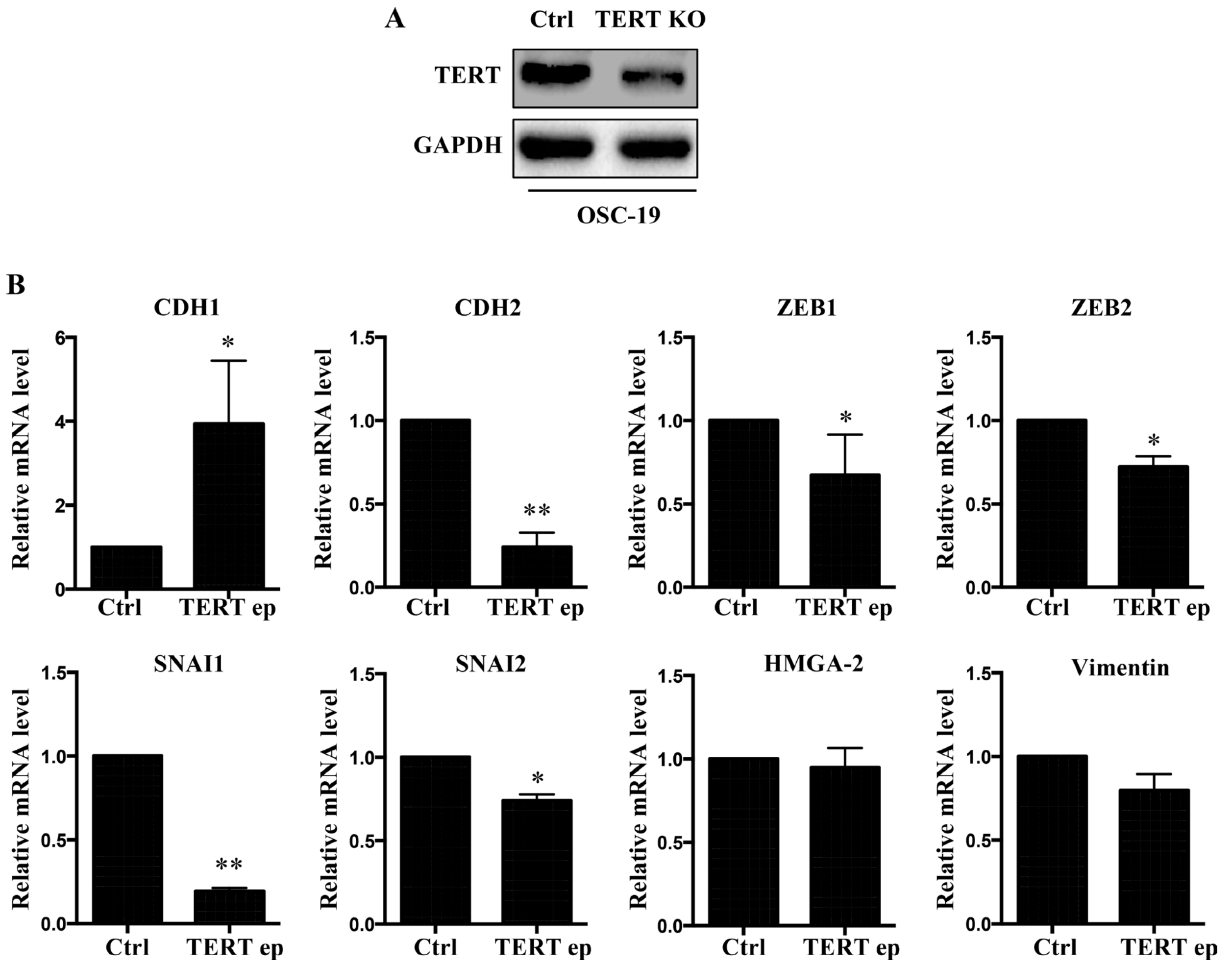

Supplementary Figure 2: Knock-out hTERT inhibits EMT in OSC-19 cells. (**A**) Knock-out hTERT was determined by Western blot. (**B**) The mRNA levels of several EMT makers in control and hTERT knockout in OSC-19 cells ($n = 3$). $^{*}P < 0.05$; $^{**}P < 0.01$.

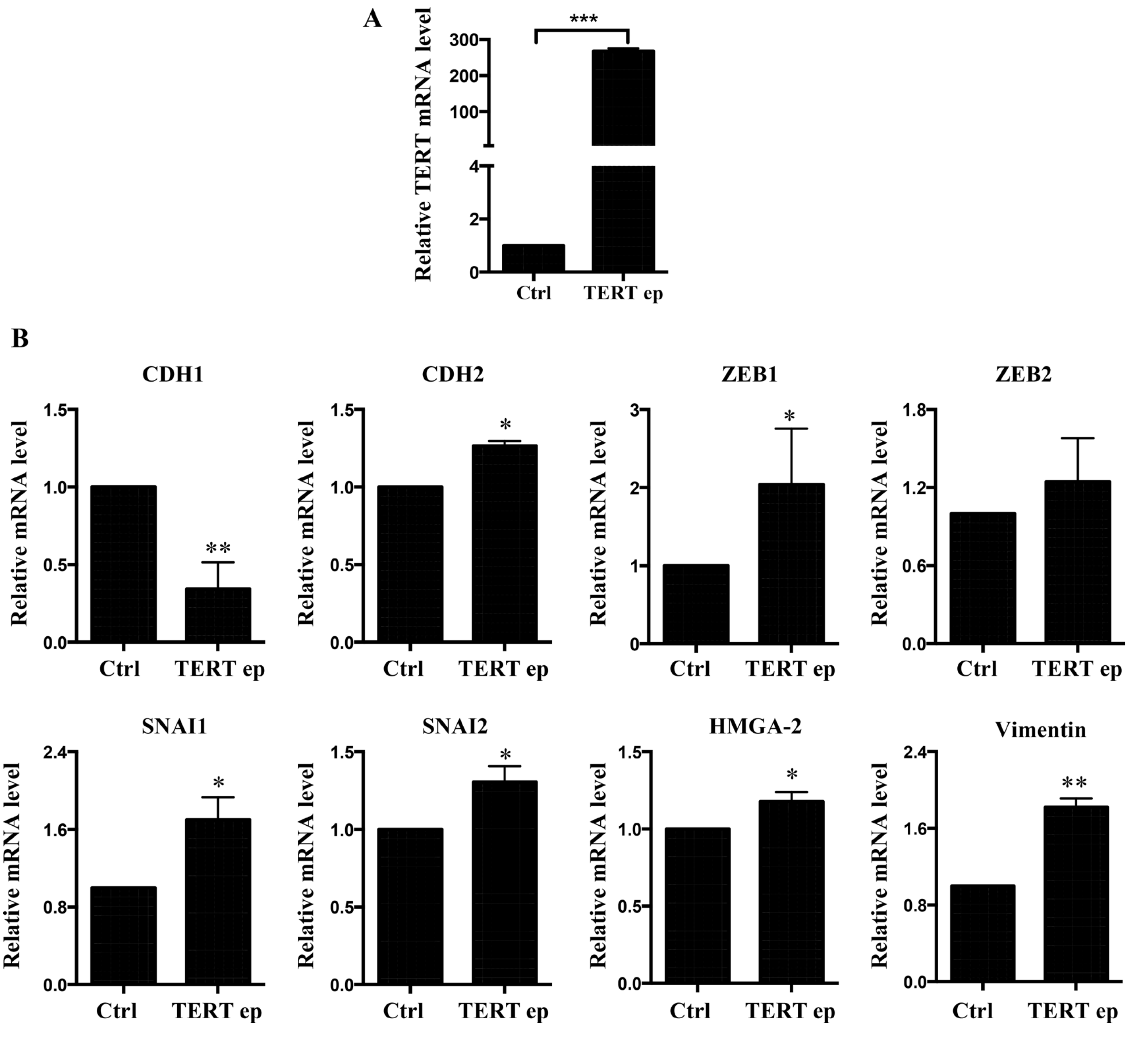

Supplementary Figure 3: Overexpression of hTERT promotes EMT in SCC-15 cells. (**A**) Over-expression of hTERT was determined by qRT-PCR. (**B**) The mRNA levels of several EMT makers in control and hTERT overexpression in SCC-15 cells ($n = 3$). *$P < 0.05$; **$P < 0.01$.

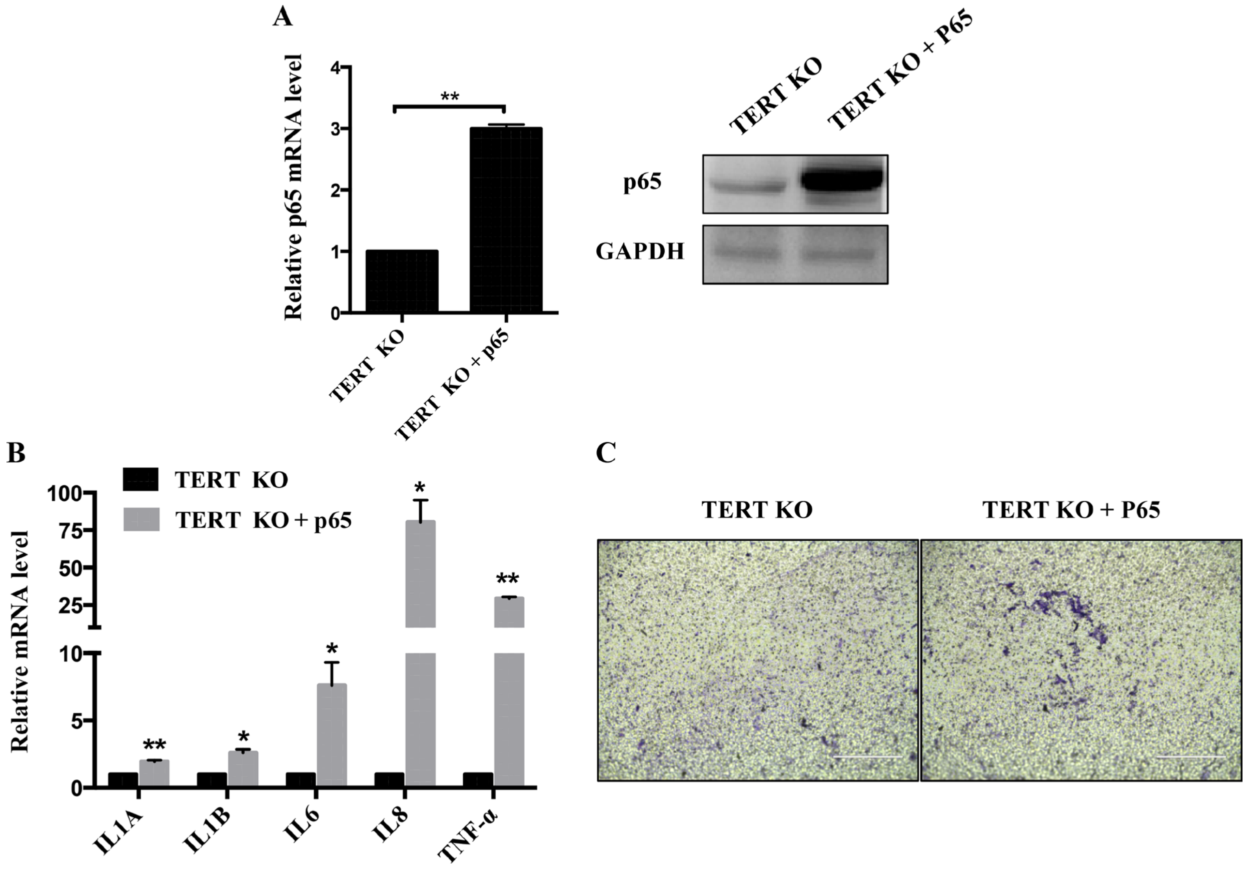

Supplementary Figure 4: Overexpreesion of p65 rescues knockout hTERT-dependent inhibition of NF-κB activation. (**A**) The mRNA and protein levels of p65 were overexpression in $hTERT^{-/-}$ cells. (**B**) The mRNA levels of several NF-κB-dependent genes were increased upon p65 overexpression in SCC-15 with hTERT knockout (n = 3). (**C**) Transwell motility assays in the $hTERT^{-/-}$ cells transfected vectors overexpressing p65. *$P < 0.05$; **$P < 0.01$.

Supplementary Table 1: Primer Sequences

Gene	Forward primer (5′-3′)	Reverse primer (5′-3′)	Melting temperature (°C)
CDH1	CGAGAGCTACACGTTCACGG	GGGTGTCGAGGGAAAAATAGG	60
CDH2	AGCCAACCTTAACTGAGGAGT	GGCAAGTTGATTGGAGGGATG	60
SNAI1	TCGGAAGCCTAACTACAGCGA	AGATGAGCATTGGCAGCGAG	60
SNAI2	CGAACTGGACACACATACAGTG	CTGAGGATCTCTGGTTGTGGT	60
ZEB1	TTACACCTTTGCATACAGAACCC	TTTACGATTACACCCAGACTGC	59
ZEB2	GGAGACGAGTCCAGCTAGTGT	CCACTCCACCCTCCCTTATTTC	60
Vimentin	GACGCCATCAACACCGAGTT	CTTTGTCGTTGGTTAGCTGGT	60
HMGA-2	ACCCAGGGGAAGACCCAAA	CCTCTTGGCCGTTTTTCTCCA	60
NF-κB	GGGCATGCGCTTCCGCTACA	TCCCCACGCTGCCTTCTTGGA	59
GAPDH	ACCACAGTCCATGCCATCAC	TCCACCACCCTGTTGCTGTA	60
IL6	ACTCACCTCTTCAGAACGAATTG	CCATCTTTGGAAGGTTCAGGTTG	59
IL8	ACTGAGAGTGATTGAGAGTGGAC	AACCCTCTGCACCCAGTTTTC	60
TNF-α	CCTCTCTCTAATCAGCCCTCTG	GAGGACCTGGGAGTAGATGAG	60
IL1A	TGGTAGTAGCAACCAACGGGA	ACTTTGATTGAGGGCGTCATTC	60
IL1B	AGCTACGAATCTCCGACCAC	CGTTATCCCATGTGTCGAAGAA	60
